# Preoperative MELD score variants and survival trajectories following in acute type A aortic dissection with follow‑up up to three years

**DOI:** 10.1186/s13019-026-04175-w

**Published:** 2026-05-11

**Authors:** Freya Sophie Jenkins, Ismail Dalyanoglu, Luis Jaime Vallejo Castano, Esma Yilmaz, Mohammed Morjan, Amin Thwairan, Johanna Wedy, Georg Ulrich Holley, Artur Lichtenberg, Hannan Dalyanoglu

**Affiliations:** 1 Department of Cardiac Surgery, Medical Faculty, Heinrich Heine University, 40225 Dusseldorf, Germany; 2 Sankt Marien Hospital Gelsenkirchen-Buer, Gelsenkirchen, 45894 Germany; 3 Cardiovascular Research Institute Düsseldorf (CARID), Medical Faculty, Heinrich Heine University, Düsseldorf, 40225 Germany

**Keywords:** Acute type A aortic dissection (ATAAD), MELD score, Charlson comorbidity index (CCI), In-hospital mortality, Long-term survival

## Abstract

**Background:**

Acute type A aortic dissection (ATAAD) remains a life-threatening cardiovascular emergency with substantial perioperative risk. Conventional cardiac risk scores often fail to capture acute multi-organ dysfunction, limiting their predictive value in emergent settings. The Model for End-Stage Liver Disease (MELD) and its variants may provide a rapid laboratory-based approach for risk stratification in ATAAD. Originally developed for hepatic disease, MELD could reflect systemic derangements relevant in ATAAD.

**Methods:**

We retrospectively analyzed 510 ATAAD patients undergoing emergency surgery (2000–2019). MELD-I, MELD-Na (sodium-adjusted), and MELD-XI (excluding INR) were calculated from admission labs and compared with the Charlson Comorbidity Index (CCI). The primary endpoints were in-hospital mortality and 3-year all-cause mortality. Statistical analyses included receiver operating characteristics (ROC) curves, DeLong tests, multivariable logistic regression, and Kaplan–Meier survival analysis (p < 0.05).

**Results:**

In-hospital mortality was 17%. MELD-I was independently associated with in-hospital mortality and showed better discriminatory performance than CCI in this cohort. (AUC 0.642 vs. 0.502; p = 0.0002). Mortality increased stepwise across MELD-I categories. Kaplan-Meier analysis demonstrated significant differences in 3-year survival (log-rank p < 0.001), with female patients showing worse outcomes (p = 0.0052).

**Conclusions:**

The MELD-I score enables rapid, lab-based assessment of acute organ dysfunction and may perform better traditional comorbidity indices for predicting early mortality in ATAAD. While its long-term prognostic value appears limited, MELD-based stratification may still provide practical support for emergency risk assessment. The observed sex-specific survival difference should be interpreted cautiously and warrants further investigation.

## Introduction

1. Epidemiology and urgency of ATAAD: Acute type A aortic dissection (ATAAD) constitutes a life-threatening cardiovascular emergency in which mortality increases by approximately 1–2% per hour following symptom onset [1, 2]. Despite advances in surgical, anesthesiologic, and perioperative management, in-hospital mortality remains high at 19–22% [3, 4]. This persistently elevated mortality highlights the need for accurate and rapid preoperative risk assessment to guide surgical decision-making, allocate perioperative resources, and improve patient counseling [5, 6].
2. Limitations of existing cardiac risk scores: Traditional cardiac risk models, such as EuroSCORE II, demonstrate moderate but inconsistent predictive accuracy in aortic surgery settings [7, 8]. Moreover, these models may overestimate mortality in high-risk or emergency cases because they were primarily developed for elective cardiac surgery [9]. Their limited applicability in ATAAD stems from the fact that they insufficiently capture acute physiological derangements characteristic of catastrophic aortic pathology.
3. Rationale for MELD in ATAAD: Given these shortcomings, attention has shifted toward risk scores that incorporate acute organ dysfunction. The Model for End-Stage Liver Disease (MELD) score—based on serum bilirubin, creatinine, and INR—was originally introduced to allocate donor livers and predict mortality in end-stage liver disease [10]. Beyond hepatology, MELD has demonstrated prognostic relevance across a variety of systemic disease states because it reflects acute hepatorenal dysfunction and coagulopathy [11, 12]. In cardiac surgery, elevated MELD scores have repeatedly been linked to increased perioperative mortality [10, 13], suggesting that MELD may be particularly suitable for emergency conditions marked by rapid multi-organ impairment such as ATAAD [14, 15].
4. Pathophysiological basis: Why MELD may work in ATAAD: ATAAD commonly induces systemic hypoperfusion, inflammatory activation, and ischemic end-organ injury. Hepatic dysfunction contributes to impaired detoxification and coagulopathy, renal hypoperfusion results in acute kidney injury, and coagulation abnormalities worsen systemic instability. Because MELD integrates bilirubin (hepatic function), creatinine (renal function), and INR (coagulation status), it captures essential elements of this acute pathophysiological cascade [11, 12]. Evidence from cardiac surgery further supports this association: postoperative mortality rises from approximately 4–5% in patients with MELD < 10 to more than 30% in those with MELD > 20 [10]. These observations indicate that MELD may reflect the real-time severity of multi-organ dysfunction more effectively than traditional scores focusing largely on chronic comorbidities.
5. The knowledge gap: Despite the biological plausibility and the proven prognostic role of MELD in other cardiovascular settings, its clinical utility in ATAAD remains insufficiently defined. Existing ATAAD risk models inadequately address acute organ dysfunction, and comparative data regarding MELD variants (MELD-I, MELD-Na, MELD-XI) versus traditional indices such as the Charlson Comorbidity Index (CCI) are sparse. Consequently, it is unclear whether MELD-based scoring improves early risk prediction or adds discriminative value beyond comorbidity-focused models. Future studies should incorporate inflammatory markers and perfusion indices to disentangle these overlapping mechanisms [16, 17].
6. Objective and hypothesis: Building on this need, we formulated the hypothesis that MELD score variants may outperform traditional comorbidity indices in predicting short-term outcomes in ATAAD.

Study Hypothesis: MELD variants outperform traditional comorbidity indices in predicting short-term outcomes in ATAAD because they quantify acute physiological derangements relevant in this emergency setting.

The study was designed with the following primary objectives:

1. To quantify the prognostic accuracy of MELD score variants (MELD-I, MELD-Na, MELD-XI) for predicting in-hospital mortality in patients presenting with acute type A aortic dissection (ATAAD).
2. To compare the discriminative performance of MELD-based models with that of the Charlson Comorbidity Index (CCI), thereby examining whether biomarker-derived indices outperform comorbidity-derived scores in acute aortic emergencies.
3. To evaluate the long-term prognostic utility of MELD score variants using structured 3-year survival follow-up data.

Although the MELD score was originally developed to assess short-term mortality in hepatic failure, accumulating evidence suggests that its constituent laboratory parameters—particularly serum creatinine and bilirubin—also reflect chronic organ vulnerability and systemic physiological reserve. These factors may continue to influence post-dissection recovery trajectories through persistent renal or hepatic dysfunction, heightened systemic inflammation, and reduced cardiometabolic resilience. Therefore, MELD-based indices may offer prognostic information that extends beyond the acute perioperative phase and contribute to long-term survival prediction in ATAAD [13].

## Materials and methods

### Study design and setting

This study was conducted as a single-center retrospective cohort analysis including 510 consecutive patients who underwent surgical repair for acute type A aortic dissection (ATAAD) at the Department of Cardiac Surgery, University Hospital Düsseldorf, between January 2000 and December 2019. The extended inclusion period was essential to assemble a sufficiently large and clinically homogeneous cohort with complete preoperative laboratory datasets, thereby ensuring adequate statistical precision for subgroup and risk-model analyses. The study was designed and reported in accordance with STROBE recommendations for observational cohort studies.

### Study population

Inclusion criteria:

- Age ≥ 18 years.
- Acute type A aortic dissection confirmed by computed tomography angiography.
- Surgical intervention **within 14 days** of symptom onset.

Exclusion criteria:

- Patients with incomplete medical records that did not allow reliable calculation of the.
- MELD score were excluded.
- Chronic or subacute aortic dissection (> 14 days from symptom onset).
- Emergency reoperations for postoperative surgical complications.

### Missing data handling

Preoperative laboratory and clinical data were available for all 510 patients included in the overall cohort analysis. However, for the one-year mortality analysis, 112 patients had to be excluded. Of these, 98 patients (19.2% of the original cohort) had incomplete laboratory records that prevented reliable calculation of MELD-I, MELD-Na, or MELD-XI scores. Specifically, missing values involved serum bilirubin, creatinine, INR, and sodium levels. Most excluded patients had missing bilirubin or INR values, whereas creatinine and sodium were less frequently unavailable. In several cases, more than one laboratory variable was missing simultaneously, making assignment to MELD risk categories impossible. Other baseline variables such as BMI, smoking status, troponin, and comorbidity data showed only low levels of missingness (< 5%) and were handled by complete-case analysis without imputation. No patients were excluded solely because of missing demographic or clinical variables. An additional 14 patients (2.7%) were excluded from the one-year mortality analysis because follow-up data were unavailable.

### Sample size considerations

Because of the retrospective design, no formal prospective sample size calculation was performed.

However, based on an anticipated in-hospital mortality of approximately 20%, a two-sided alpha level of 0.05, and an odds ratio of at least 1.5 for MELD-based predictors, a cohort size of 510 patients provides more than 80% statistical power to detect clinically meaningful associations reported in contemporary ATAAD cohorts [5, 6]. With 85 in-hospital deaths, the number of events was also sufficient to support multivariable logistic regression while maintaining an acceptable events-per-variable ratio. This justification reflects the expected precision of effect estimates rather than a priori sample calculation [18].

### Variable definitions

Predictor variables:

- **MELD-I**: 9.57 × ln(creatinine) + 3.78 × ln(bilirubin) + 11.20 × ln(INR) + 6.43.
- **MELD-Na**: MELD-I + 1.59 × (135 – sodium).
- **MELD-XI**:5.11 × ln(bilirubin) + 11.76 × ln(creatinine) + 9.44.

(variant excluding INR, frequently used in anticoagulated patients)

- **Charlson Comorbidity Index (CCI)**: standard and age-adjusted formulations.
- **Demographics and Baseline Variables**: age, sex, BMI, hypertension, diabetes, dyslipidemia, smoking, ASA class.
- **Laboratory Variables**: troponin (high-sensitivity assay; institution-specific reference), bilirubin, creatinine, INR, sodium.

Justification of MELD Variants:

MELD-I was selected as the primary risk model due to its integration of hepatic, renal, and coagulative biomarkers—key organ systems affected during ATAAD-related malperfusion.

To account for physiological variability relevant in acute aortic syndromes, MELD-Na (incorporating sodium as an electrolyte disturbance marker) and MELD-XI (excluding INR for patients with unreliable coagulation parameters due to anticoagulation) were analyzed as complementary models.

These variants enable organ-specific refinement of risk prediction and are fully applicable in the preoperative setting, where rapid risk stratification is crucial. While alternative ICU-based scores such as SOFA or SAPS II also assess organ failure, these are often applied in postoperative or intensive care contexts. In contrast, MELD variants were specifically chosen for their preoperative applicability using admission laboratory data, which is crucial for early triage in ATAAD. The rapid availability of MELD components makes them suitable for urgent settings, though turnaround times may vary between institutions.

### Outcome variables

Primary Endpoints

- In-hospital mortality.
- 3-year all-cause mortality.

One-year vital status was obtained via structured outpatient follow-up, electronic medical records, and—when required—telephone contact.

Secondary Endpoint

- 1-year all-cause mortality.

One‑year mortality was explored as a secondary exploratory outcome for intermediate‑term contextualization.

- Total length of hospital stay (LOS) (defined as days from admission to discharge; LOS tertiles were derived from cohort-specific distribution).

One-year vital status was obtained via structured outpatient follow-up, electronic medical records, and when required telephone contact.

### Statistical analysis

Continuous variables were reported as medians with interquartile ranges, and categorical variables as absolute and relative frequencies. Normality of continuous variables was assessed using the Shapiro–Wilk test. Group comparisons were performed using the Mann–Whitney U test or Student’s t-test (depending on distribution) and χ² tests for categorical variables. Discriminatory performance of MELD variants and the CCI was evaluated using receiver operating characteristic (ROC) analysis. To evaluate model robustness, internal validation was performed using bootstrap resampling with 1000 iterations. Corrected AUC values remained similar to the original estimates, indicating stable discriminatory performance despite the absence of an external validation cohort. Comparisons between AUCs were performed using the DeLong method. Optimal cut-off values were derived via Youden’s index. Multivariable logistic regression was conducted to identify independent predictors of in-hospital mortality. Collinearity was assessed using variance inflation factors, which are reported in the Results section. Given the exploratory nature of the analysis, stepwise variable selection was used cautiously and interpreted in the context of overall model stability. Long-term survival was analyzed using Kaplan–Meier curves and compared via log-rank tests. To reduce the risk of overfitting, the number of variables included in the multivariable model was restricted according to the number of outcome events, maintaining approximately one predictor per 10 events. Stepwise variable selection was used only as an exploratory approach and interpreted cautiously. Internal model robustness was additionally assessed by bootstrap resampling with 1000 iterations. All analyses were performed using R version 4.3.2 (R Foundation for Statistical Computing, Vienna, Austria). Statistical significance was defined as *p* < 0.05.

### Ethics

The study was approved by the local ethics committee (Heinrich Heine University Düsseldorf, Protocol #2023–2566, December 6, 2023) and conducted in accordance with the Declaration of Helsinki. Due to the retrospective and anonymized design, patient consent was waived.

## Results

### Baseline characteristics

The study cohort comprised 510 patients with median age 63 years (IQR 54–72) and male predominance (66%, *n* = 338). Substantial comorbidity burden was evident with hypertension in 62%, hyperlipidemia in 22%, and diabetes in 7.6% of patients. Median BMI was 26.9 kg/m² (IQR 24.5–29.7) (Table 1).

**Table 1:** Baseline characteristics all patients

Characteristic	*n* = 510^1^
Male sex	338 (66%)
BMI^2^	26.9 (24.5, 29.7)
Age in years	63 (54, 72)
History of myocardial infarction^3^	48 (9.4%)
Ever-smoker^4^	108 (21%)
Diabetes	39 (7.6%)
Hyperlipidemia	113 (22%)
Hypertension^5^	316 (62%)
Previous cardiac surgery	24 (4.7%)
Active endocarditis	2 (0.4%)
Marfan syndrome	4 (0.8%)
Preoperative troponin levels^6^	25 (12, 55)
Entry zone	
E1^7^	475 (93%)
E2^8^	32 (6.3%)
E3^9^	3 (0.6%)
Malperfusion class	
M0^10^	344 (67%)
M1^11^	4 (0.8%)
M2^12^	143 (28%)
M3^13^	19 (3.7%)
Total arch replacement	332 (65%)
Duration of surgery in minutes^14^	334 (272, 413)
In-hospital mortality rate^15^	85 (17%)
Length of hospital stay^16^	14 (9, 21)
Charlson Comorbidity Score, including age^17^	4.00 (3.00, 5.00)
MELD-I^18^	9.1 (6.2, 12.6)
MELD-Na^19^	9.6 (6.4, 13.2)
MELD-XI^20^	8.2 (5.4, 10.8)
MELD-I group^21^	
1	286 (56%)
2	164 (32%)
3	60 (12%)
4	0 (0%)
MELD-Na group^22^	
1	266 (52%)
2	179 (35%)
3	65 (13%)
MELD-XI group^23^	
1	355 (70%)
2	148 (29%)
3	7 (1.4%)
One-year mortality rate^24^	119 (30%)

^1^n, ^2^body mass index, ^3^myocardial infarction in past medical history, ^4^smoking active or in past medical history, ^5^arterial hypertension, ^6^in nanograms per liter (min, max), ^7^E1 = entry tear located in the ascending aorta, ^8^E2 = entry tear located in the aortic arch, ^9^E3 = entry tear located in the descending thoracic aorta, ^10^M0 = no malperfusion, ^11^M1 = coronary malperfusion, ^12^M2 = visceral or renal, malperfusion, ^13^M3 = peripheral or multiple-site malperfusion, ^14^in minutes, ^15i^in days, ^16^ in days, ^17^unadjusted Charlson Comorbidity Index (CCI) in absolute CCI points, ^18^Model for End-Stage Liver Disease (MELD-I), ^19^MELD including sodium levels (MELD-Na), ^21^MELD excluding INR, ^21^MELD-I, ^22^MELD-Na, and ^23^MELD-XI were each grouped into three risk categories: scores < 9 = low risk, scores 9–16 = intermediate risk and scores > 17 = high risk. EuroSCORE II and STS scores could not be calculated due to incomplete documentation of required variables in this emergency setting, ^24^(%),

### Primary outcome: in-hospital mortality

In-hospital mortality occurred in 85 patients (16.7%). Non-survivors were significantly older (median 68 vs. 62 years, *p* < 0.001) and had higher rates of prior myocardial infarction (19% vs. 7.5%, *p* = 0.001). MELD-I scores were markedly higher among non-survivors (median 11.8 vs. 8.5, *p* < 0.001) (Table 2).

**Table 2:** Characteristics by in-hospital mortality

Variable	Overall *n* = 510^1^	Survivors *n* = 425^1^	Non-survivors *n* = 85^1^	*p*-value^2^
Male sex	338 (66%)	291 (68%)	47 (55%)	0.019
BMI^3^	26.9 (24.5, 29.7)	26.9 (24.5, 29.7)	26.6 (24.7, 29.4)	0.9
Age in years^4^	63 (54, 72)	62 (54, 72)	68 (60, 76)	< 0.001
History of MI^5^	48 (9.4%)	32 (7.5%)	16 (19%)	0.001
Ever-smoker^6^	108 (21%)	98 (23%)	10 (12%)	0.020
Diabetes	39 (7.6%)	31 (7.3%)	8 (9.4%)	0.5
Hyperlipidemia	113 (22%)	91 (21%)	22 (26%)	0.4
Hypertension^7^	316 (62%)	256 (60%)	60 (71%)	0.073
Previous cardiac surgery^8^	24 (4.7%)	18 (4.2%)	6 (7.1%)	0.3
Active endocarditis	2 (0.4%)	1 (0.2%)	1 (1.2%)	0.3
Marfan syndrome	4 (0.8%)	3 (0.7%)	1 (1.2%)	0.5
Preoperative troponin^9^	25 (12, 55)	22 (11, 55)	26 (15, 65)	0.034
Entry zone				0.017
E1^10^	475 (93%)	401 (94%)	74 (87%)	
E2^11^	32 (6.3%)	23 (5.4%)	9 (11%)	
E3^12^	3 (0.6%)	1 (0.2%)	2 (2.4%)	
Malperfusion class				0.2
M0^13^	344 (67%)	283 (67%)	61 (72%)	
M1^14^	4 (0.8%)	2 (0.5%)	2 (2.4%)	
M2^15^	143 (28%)	124 (29%)	19 (22%)	
M3^16^	19 (3.7%)	16 (3.8%)	3 (3.5%)	
Total arch replacement	332 (65%)	278 (65%)	54 (64%)	0.7
Surgery duration in mins^17^	334 (272, 413)	323 (265, 396)	397 (324, 478)	< 0.001
Hospital stay in days^18^	14 (9, 21)	15 (11, 22)	3 (1, 10)	< 0.001
Euroscore II	0 (NA%)	0 (NA%)	0 (NA%)	
CCCI with age^19^	4.00 (3.00, 5.00)	4.00 (3.00, 5.00)	5.00 (4.00, 6.00)	< 0.001
MELD-I1^20^	9.1 (6.2, 12.6)	8.5 (6.0, 11.8)	11.8 (7.6, 16.8)	< 0.001
MELD-Na^21^	9.6 (6.4, 13.2)	9.3 (6.2, 12.4)	12.1 (8.2, 17.2)	< 0.001
MELD-XI^22^	8.2 (5.4, 10.8)	8.0 (5.4, 10.5)	9.2 (6.8, 11.6)	0.006
MELD-I group^23^				< 0.001
1	286 (56%)	254 (60%)	32 (38%)	
2	164 (32%)	126 (30%)	38 (45%)	
3	60 (12%)	45 (11%)	15 (18%)	
MELD-Na group^24^				0.001
1	266 (52%)	237 (56%)	29 (34%)	
2	179 (35%)	139 (33%)	40 (47%)	
3	65 (13%)	49 (12%)	16 (19%)	
MELD-XI group^25^				0.022
1	355 (70%)	306 (72%)	49 (58%)	
2	148 (29%)	114 (27%)	34 (40%)	
3	7 (1.4%)	5 (1.2%)	2 (2.4%)	

^1^n; Median (Q1, Q3), ^2^Pearson’s Chi-squared test; Wilcoxon rank sum test; Fisher’s exact test, ^3^body mass index, ^4^in years, ^5^myocardial infarction in past medical history, ^6^smoking active or in past medical history, ^7^arterial hypertension, ^8^previous cardiac surgery, ^9^in nanograms per liter, ^10^E1 = entry tear located in the ascending aorta, ^11^E2 = entry tear located in the aortic arch, ^12^E3 = entry tear located in the descending thoracic aorta, ^13^M0 = no malperfusion, ^14^M1 = coronary malperfusion, ^15^M2 = visceral or renal, malperfusion, ^16^M3 = peripheral or multiple-site malperfusion, ^17^in minutes, ^18^in days, ^19^unadjusted Charlson Comorbidity Index (CCI) in absolute CCI points, ^20^Model for End-Stage Liver Disease (MELD-I), ^21^MELD including sodium levels (MELD-Na), ^22^MELD excluding INR, ^23^MELD-I, ^24^MELD-Na, and ^25^MELD-XI were each grouped into three risk categories: scores < 9 = low risk, scores 9–16 = intermediate risk and scores > 17 = high risk. EuroSCORE II and STS scores could not be calculated due to incomplete documentation of required variables in this emergency setting, 5cardiopulmonary resuscitation in past medical history, 6myocardial infarction in past medical history, 7smoking active or in past medical history, 8arterial hypertension, 9ASA physical status classification system, 10in millimeters, 11previous cardiac surgery, 12in nanograms per liter, 13in minutes, 14in days, 15unadjusted Charlson Comorbidity Index (CCI) in absolute points, 16age-adjusted CCI, in absolute CCI points, 17Model for End-Stage Liver Disease (MELD), 18MELD including sodium levels (MELD-Na), 19MELD excluding INR, ), 20MELD I, MELD-Na, and MELD-XI were each grouped into three risk categories: scores < 10 = group 1, scores 10–19 = group 2, and scores > 19 = group 3

### Long-term survival analysis with kaplan–meier estimates (up to 3 years)

**Fig. 1:**
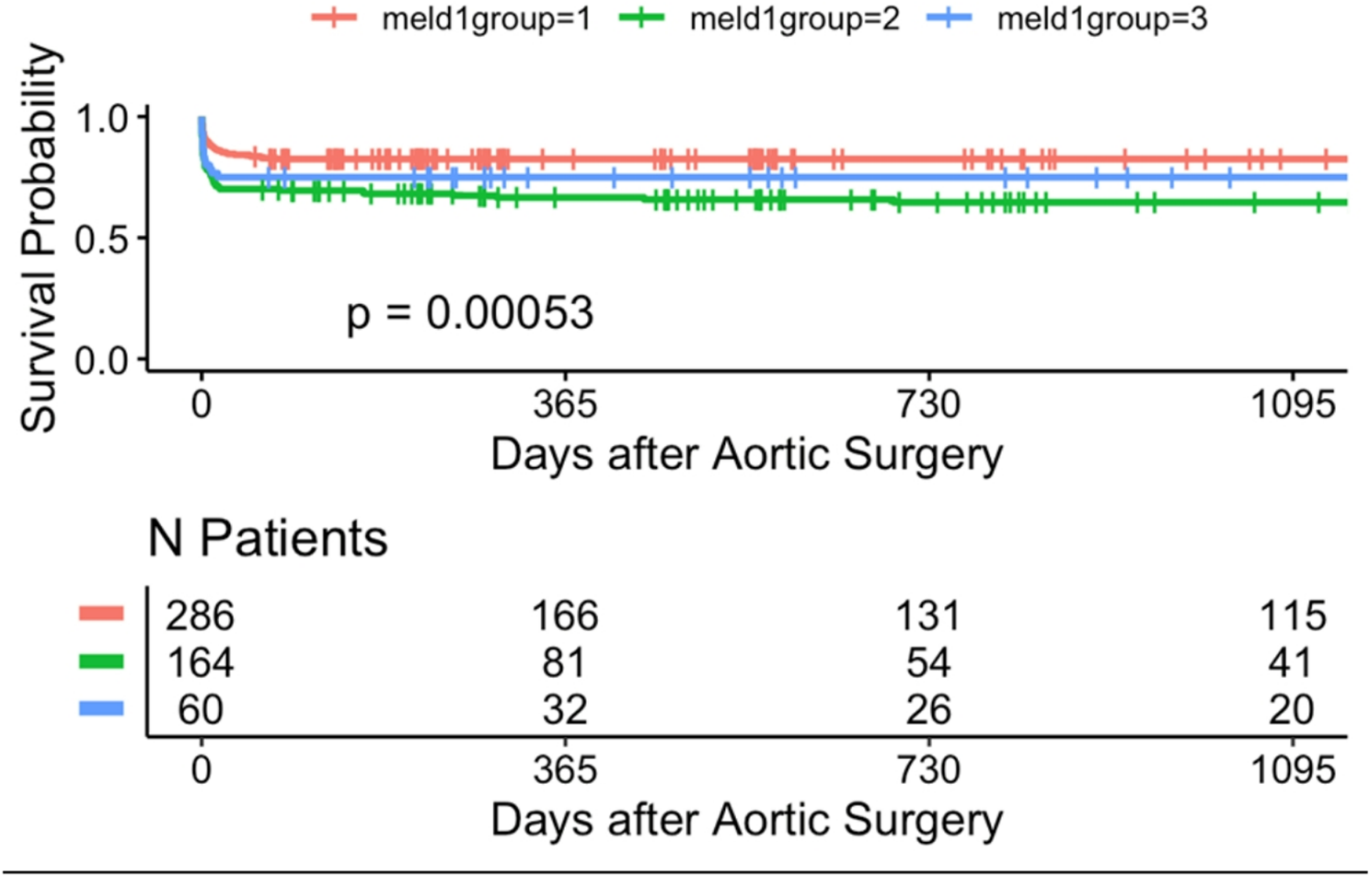
Kaplan-Meier survival analyses (curtailed at 3 years) for Meld 1

**Fig. 2:**
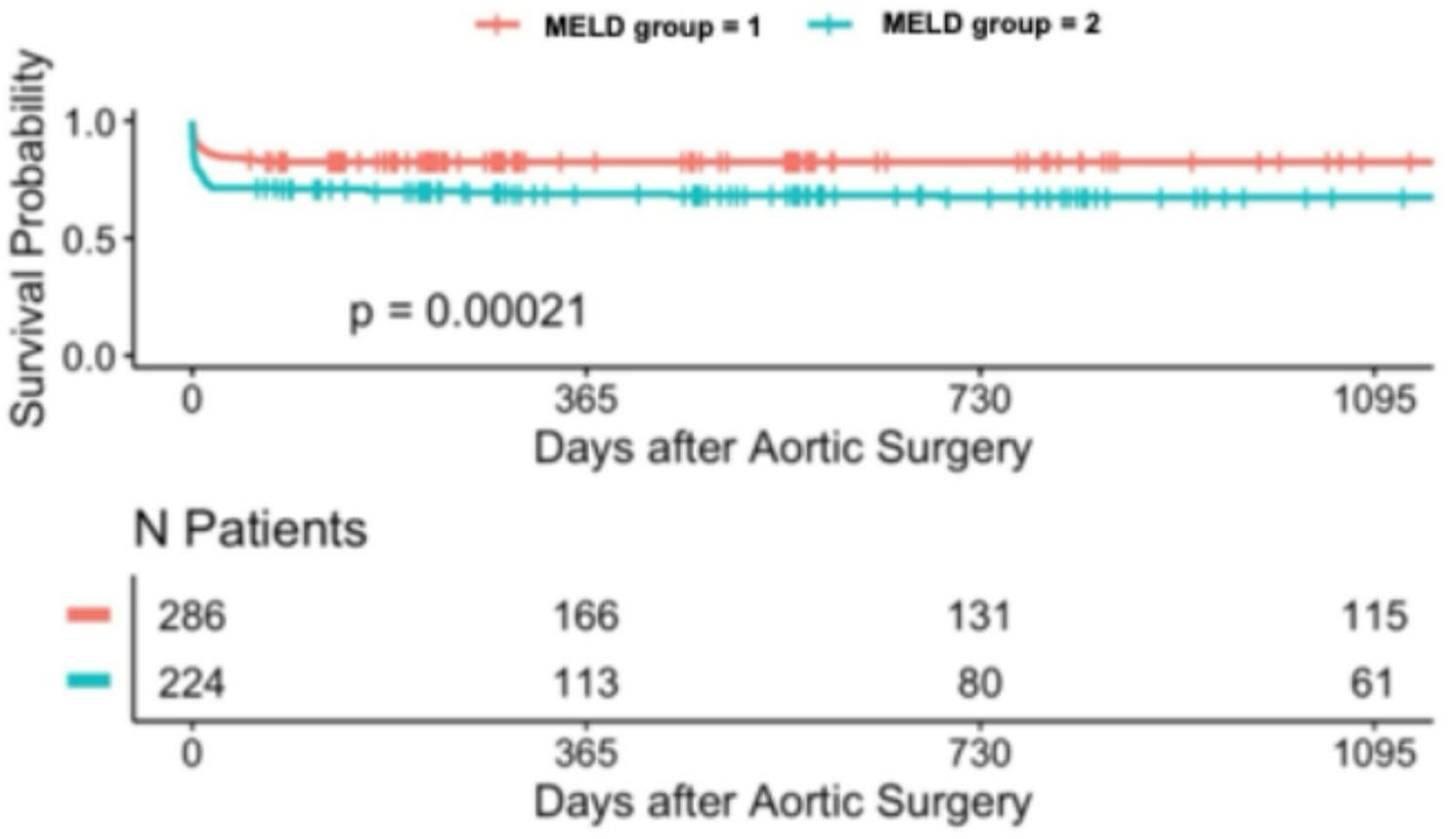
Kaplan-Meier survival analyses (curtailed at 3 years) for MELD-I with different levels

**Fig. 3:**
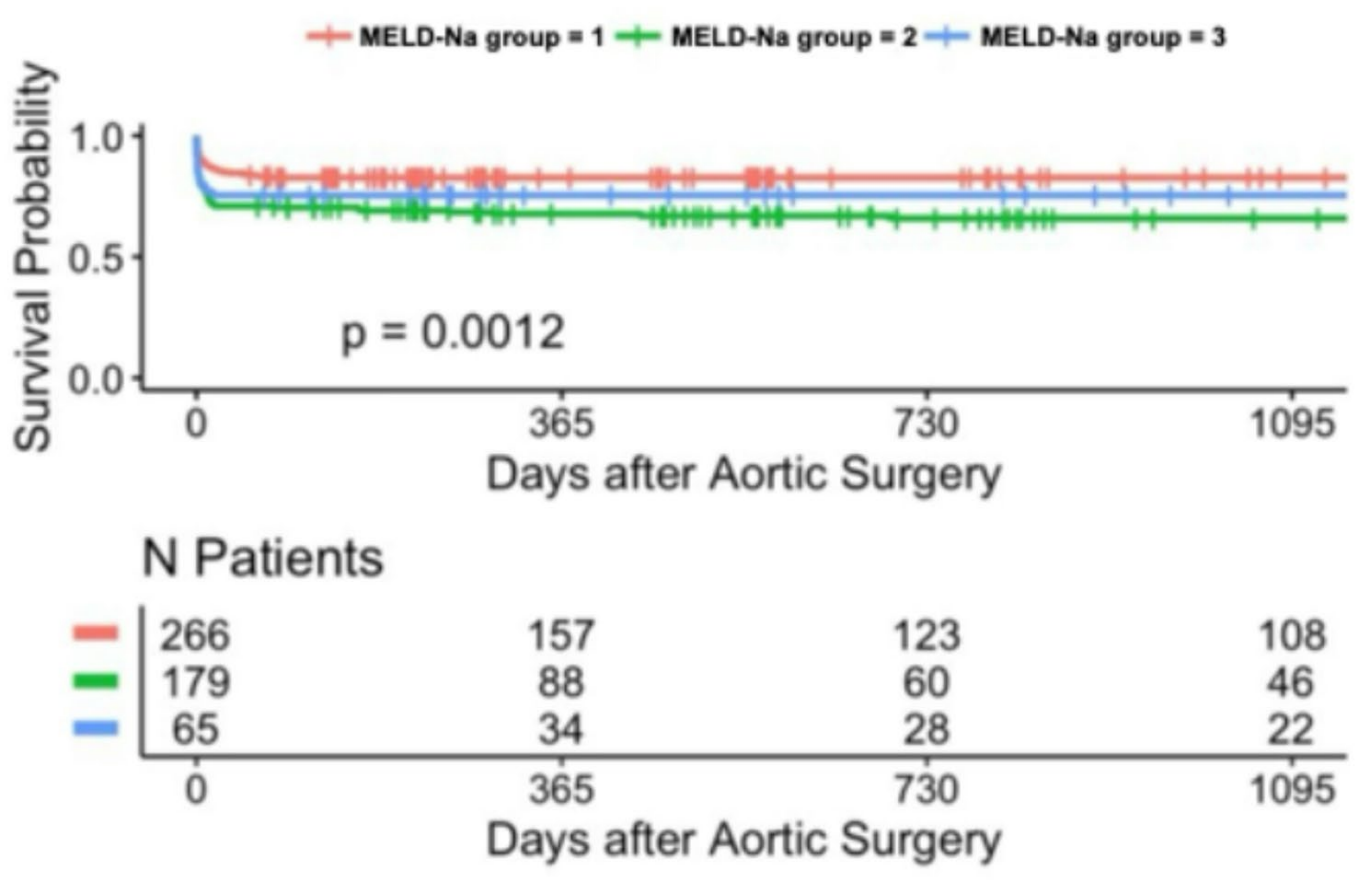
Kaplan-Meier survival analyses (curtailed at 3 years) for MELD-Na with different levels

**Fig. 4:**
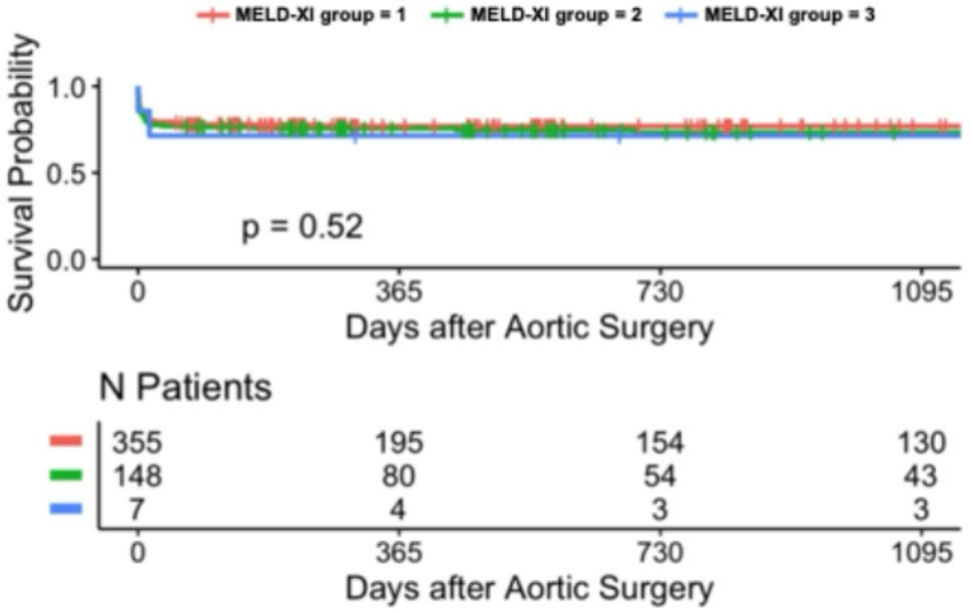
Kaplan-Meier survival analyses (curtailed at 3 years) for MELD-XI with different levels

**Fig. 5:**
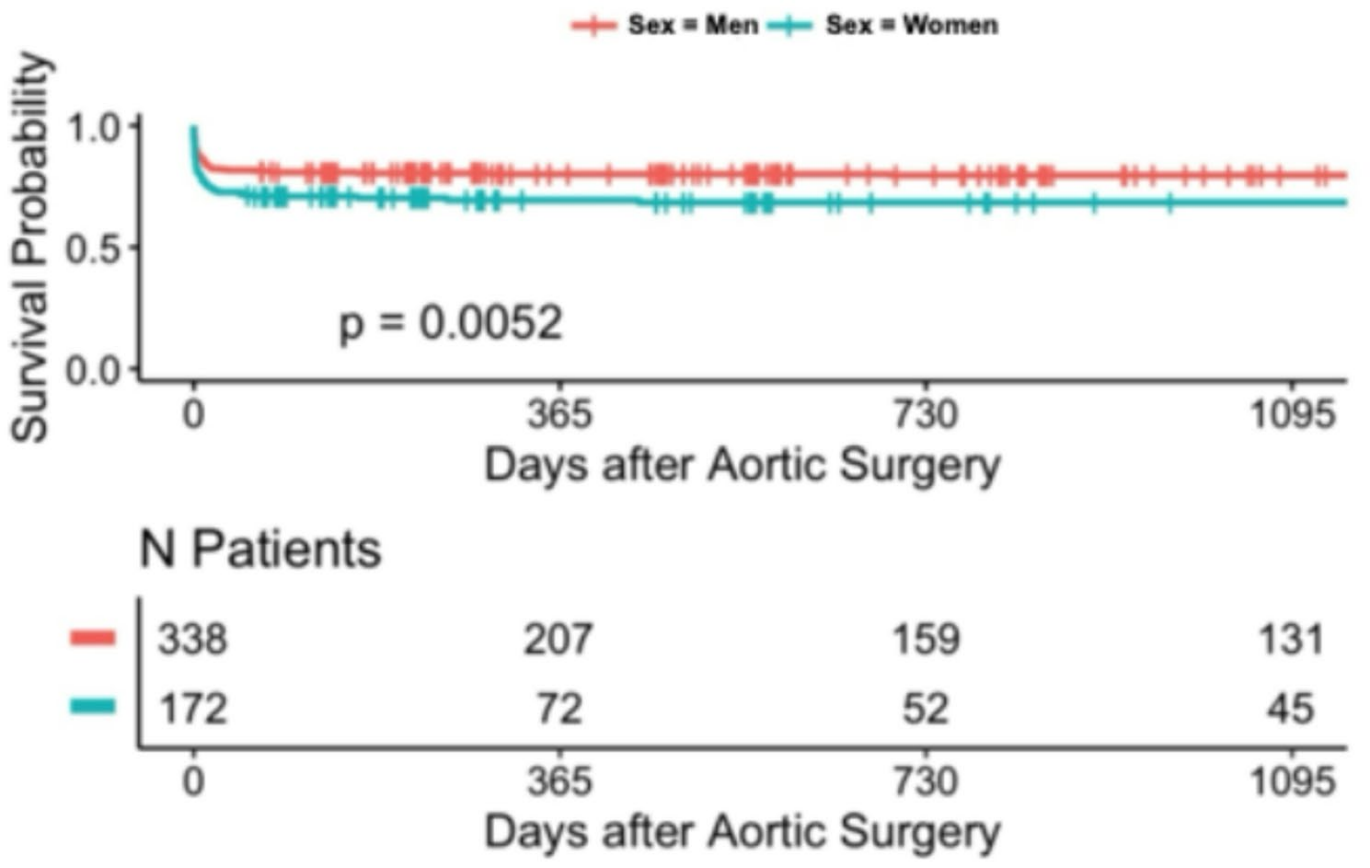
Kaplan-Meier survival analyses for Sex (curtailed at 3 years)

**Fig. 6:**
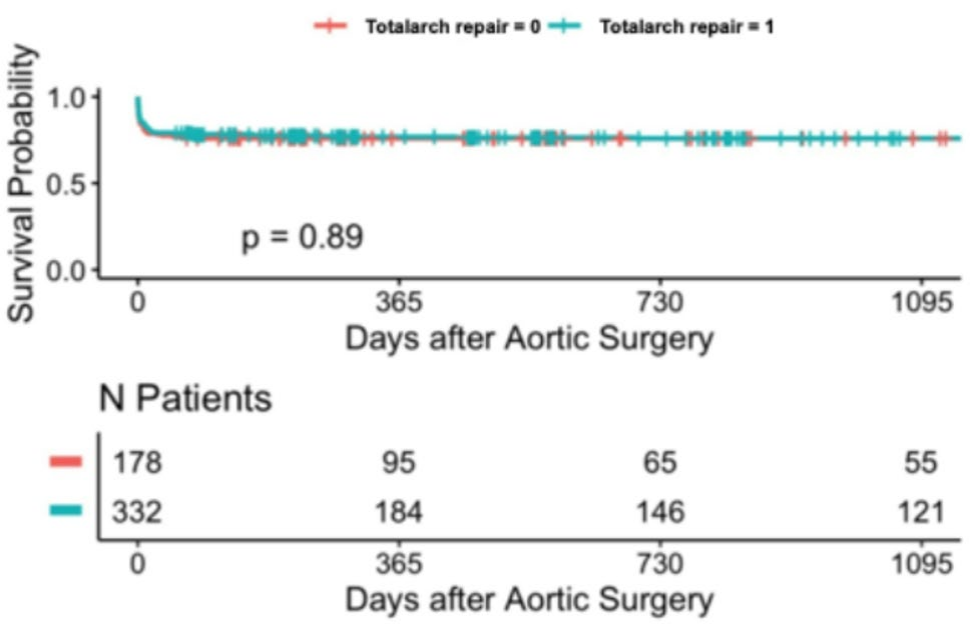
Kaplan-Meier survival analyses for need of aortic total-arch replacement (curtailed at 3 years)

During the follow-up period, long-term survival was additionally evaluated. Kaplan-Meier analysis demonstrated significant differences in long-term survival across MELD-I risk groups over the 3-year follow-up period in patients undergoing surgery for acute type A aortic dissection. Stratification according to MELD score variants revealed significant prognostic separation: 3-year survival rates were: 40.2% (*n* = 254, low-risk MELD-I < 9), 25% (*n* = 126, intermediate 9–16), and 33.3% (*n* = 45, high-risk ≥ 17) (*p* < 0.001) (Tables 3 and 4). Notably, the high-risk MELD-I group (score ≥ 17) showed a 3-year survival rate of around 33.3.% (*p* < 0.001). Similar trends were observed with MELD-Na and MELD-XI scores. In contrast, stratification by the Charlson Comorbidity Index showed less pronounced separation in long-term survival. These findings suggest that markers of acute hepatic and renal dysfunction, as well as coagulation abnormalities, may contribute to early and long-term risk stratification, as captured by the MELD scores, for both short- and long-term outcomes in this patient population (Figs. 1, 2, 3, 4, 5 and 6).

**Table 3:** Characteristics by length of stay group

Variable	Overall *n* = 510^1^	< 14 days *n* = 277^1^	> 14 days *n* = 233^1^	*p*-value^2^
Male sex	338 (66%)	182 (66%)	156 (67%)	0.8
BMI^3^	26.9 (24.5, 29.7)	26.5 (24.2, 29.4)	27.4 (24.7, 30.3)	0.024
Age in years^4^	63 (54, 72)	62 (55, 73)	63 (54, 72)	0.5
History of MI^5^	48 (9.4%)	29 (10%)	19 (8.2%)	0.4
Ever-smoker^6^	108 (21%)	51 (18%)	57 (24%)	0.10
Diabetes	39 (7.6%)	18 (6.5%)	21 (9.0%)	0.3
Hyperlipidemia	113 (22%)	64 (23%)	49 (21%)	0.6
Hypertension^7^	316 (62%)	167 (60%)	149 (64%)	0.4
Previous cardiac surgery^8^	24 (4.7%)	9 (3.2%)	15 (6.4%)	0.090
Active endocarditis	2 (0.4%)	2 (0.7%)	0 (0%)	0.5
Marfan syndrome	4 (0.8%)	1 (0.4%)	3 (1.3%)	0.3
Preoperative troponin^9^	25 (12, 55)	24 (12, 46)	25 (11, 58)	0.3
Entry zone				0.5
E1^10^	475 (93%)	261 (94%)	214 (92%)	
E2^11^	32 (6.3%)	14 (5.1%)	18 (7.7%)	
E3^12^	3 (0.6%)	2 (0.7%)	1 (0.4%)	
Malperfusion class				0.079
M0^13^	344 (67%)	200 (72%)	144 (62%)	
M1^14^	4 (0.8%)	2 (0.7%)	2 (0.9%)	
M2^15^	143 (28%)	66 (24%)	77 (33%)	
M3^16^	19 (3.7%)	9 (3.2%)	10 (4.3%)	
Total arch replacement	332 (65%)	183 (66%)	149 (64%)	0.6
Surgery duration in mins^17^	334 (272, 413)	327 (262, 411)	345 (280, 415)	0.2
Hospital stay in days^18^	14 (9, 21)	9 (3, 12)	22 (18, 33)	< 0.001
CCI including age^19^	4.00 (3.00, 5.00)	4.00 (3.00, 5.00)	4.00 (3.00, 5.00)	0.5
MELD-I^20^	9.1 (6.2, 12.6)	8.9 (6.4, 12.1)	9.4 (5.8, 13.4)	> 0.9
MELD-Na^21^	9.6 (6.4, 13.2)	9.6 (6.7, 12.9)	9.6 (6.1, 14.1)	0.8
MELD-XI^22^	8.2 (5.4, 10.8)	8.0 (5.2, 10.5)	8.3 (5.7, 11.3)	0.12
MELD-I group^23^				0.092
1	286 (56%)	157 (57%)	129 (55%)	
2	164 (32%)	95 (34%)	69 (30%)	
3	60 (12%)	25 (9.0%)	35 (15%)	
MELD-Na group^24^				0.2
1	266 (52%)	145 (52%)	121 (52%)	
2	179 (35%)	103 (37%)	76 (33%)	
3	65 (13%)	29 (10%)	36 (15%)	
MELD-XI group^25^				0.4
1	355 (70%)	200 (72%)	155 (67%)	
2	148 (29%)	74 (27%)	74 (32%)	
3	7 (1.4%)	3 (1.1%)	4 (1.7%)	

^1^n; Median (Q1, Q3), ^2^Pearson’s Chi-squared test; Wilcoxon rank sum test; Fisher’s exact test, ^3^body mass index, ^4^in years, ^5^myocardial infarction in past medical history, ^6^smoking active or in past medical history, ^7^arterial hypertension, ^8^previous cardiac surgery, ^9^in nanograms per liter, ^10^E1 = entry tear located in the ascending aorta, ^11^E2 = entry tear located in the aortic arch, ^12^E3 = entry tear located in the descending thoracic aorta, ^13^M0 = no malperfusion, ^14^M1 = coronary malperfusion, ^15^M2 = visceral or renal, malperfusion, ^16^M3 = peripheral or multiple-site malperfusion, ^17^in minutes, ^18^in days, ^19^unadjusted Charlson Comorbidity Index (CCI) in absolute CCI points, ^20^Model for End-Stage Liver Disease (MELD-I), ^21^MELD including sodium levels (MELD-Na), ^22^MELD excluding INR, ^23^MELD-I, ^24^MELD-Na, and ^25^MELD-XI were each grouped into three risk categories: scores < 9 = low risk, scores 9–16 = intermediate risk and scores > 17 = high risk. EuroSCORE II and STS scores could not be calculated due to incomplete documentation of required variables in this emergency setting,

**Table 4:** Characteristics by MELD1 group

Variable	Overall *n* = 510^1^	Group 1 *n* = 286^1^	Group 2 *n* = 164^1^	Group 3 *n* = 60^1^	*p*-value^2^
Male sex	338 (66%)	168 (59%)	125 (76%)	45 (75%)	< 0.001
BMI^3^	26.9 (24.5, 29.7)	26.6 (23.9, 29.4)	26.9 (24.8, 29.5)	27.7 (25.9, 31.1)	0.029
Age in years^4^	63 (54, 72)	63 (53, 72)	62 (55, 74)	64 (56, 74)	0.4
History of MI^5^	48 (9.4%)	21 (7.3%)	17 (10%)	10 (17%)	0.070
Ever-smoker^r6^	108 (21%)	65 (23%)	33 (20%)	10 (17%)	0.5
Diabetes	39 (7.6%)	24 (8.4%)	7 (4.3%)	8 (13%)	0.055
Hyperlipidemia	113 (22%)	65 (23%)	36 (22%)	12 (20%)	0.9
Hypertension^7^	316 (62%)	181 (63%)	96 (59%)	39 (65%)	0.5
Active endocarditis	2 (0.4%)	1 (0.3%)	1 (0.6%)	0 (0%)	> 0.9
Marfan syndrome	4 (0.8%)	2 (0.7%)	1 (0.6%)	1 (1.7%)	0.6
Preop troponin^8^	25 (12, 55)	19 (10, 44)	27 (14, 62)	33 (17, 112)	< 0.001
Entry zone					0.5
E1^9^	475 (93%)	268 (94%)	152 (93%)	55 (92%)	
E2^10^	32 (6.3%)	16 (5.6%)	12 (7.3%)	4 (6.7%)	
E3^11^	3 (0.6%)	2 (0.7%)	0 (0%)	1 (1.7%)	
Malperfusion class					0.3
M0^12^	344 (67%)	202 (71%)	102 (62%)	40 (67%)	
M1^13^	4 (0.8%)	2 (0.7%)	1 (0.6%)	1 (1.7%)	
M2^14^	143 (28%)	75 (26%)	52 (32%)	16 (27%)	
M3^15^	19 (3.7%)	7 (2.4%)	9 (5.5%)	3 (5.0%)	
Arch replacement	332 (65%)	185 (65%)	109 (66%)	38 (63%)	0.9
Surgery duration^16^	334 (272, 413)	332 (264, 410)	341 (276, 430)	325 (286, 407)	0.5
In-hospital death^17^	85 (17%)	32 (11%)	38 (23%)	15 (25%)	< 0.001
Hospital stay^18^	14 (9, 21)	14 (10, 20)	13 (7, 21)	18 (10, 27)	0.10
CCI with age^19^	4.00 (3.00, 5.00)	4.00 (3.00, 5.00)	4.00 (3.00, 5.00)	5.00 (4.00, 6.00)	0.024
MELD-I^20^	9.1 (6.2, 12.6)	6.4 (5.1, 8.1)	11.9 (10.7, 14.2)	21.7 (21.4, 25.0)	< 0.001
MELD-Na^21^	9.6 (6.4, 13.2)	6.5 (5.4, 8.4)	12.3 (10.7, 15.1)	22.4 (21.6, 26.3)	< 0.001
MELD-XI^22^	8.2 (5.4, 10.8)	7.0 (4.8, 9.0)	10.0 (7.6, 12.5)	9.5 (6.1, 14.5)	< 0.001
MELD-Na group^23^					< 0.001
1	266 (52%)	266 (93%)	0 (0%)	0 (0%)	
2	179 (35%)	20 (7.0%)	159 (97%)	0 (0%)	
3	65 (13%)	0 (0%)	5 (3.0%)	60 (100%)	
MELD-XI group^24^					< 0.001
1	355 (70%)	241 (84%)	82 (50%)	32 (53%)	
2	148 (29%)	45 (16%)	81 (49%)	22 (37%)	
3	7 (1.4%)	0 (0%)	1 (0.6%)	6 (10%)	
MELD-I group					
In-hospital mortality^25^	510	32 (11%)	38 (23%)	15 (25%)	< 0.001
One-year mortality^26^	119 (30%)	50 (23%)	54 (40%)	15 (32%)	0.003
3-year survival^27^		254 (40.2%)	126 (25.0%)	45 (33.3%)	< 0.001
Hospital stay group					0.092
< 14 days	277 (54%)	157 (55%)	95 (58%)	25 (42%)	
> 14 days	233 (46%)	129 (45%)	69 (42%)	35 (58%)	

^1^n; Median (Q1, Q3), ^2^Pearson’s Chi-squared test; Wilcoxon rank sum test; Fisher’s exact test, ^3^body mass index, ^4^in years, ^5^myocardial infarction in past medical history, ^6^smoking active or in past medical history, ^7^arterial hypertension, ^8^in nanograms per liter, ^9^E1 = entry tear located in the ascending aorta, ^10^E2 = entry tear located in the aortic arch, ^11^E3 = entry tear located in the descending thoracic aorta, ^12^M0 = no malperfusion, ^13^M1 = coronary malperfusion, ^14^M2 = visceral or renal, malperfusion, ^15^M3 = peripheral or multiple-site malperfusion, ^16^in minutes, ^17^ (%), ^18^in days, ^19^unadjusted Charlson Comorbidity Index (CCI) in absolute CCI points, ^20^Model for End-Stage Liver Disease (MELD-I), ^21^MELD including sodium levels (MELD-Na), ^22^MELD excluding INR, ^2^MELD-Na, and ^24^MELD-XI were each grouped into three risk categories: scores < 9 = low risk, scores 9–16 = intermediate risk and scores > 17 = high risk. EuroSCORE II and STS scores could not be calculated due to incomplete documentation of required variables in this emergency setting, ^25^n(%), ^26^n(%), ^27^n(%)

### MELD score performance

Risk Stratification Categories:

MELD-I risk stratification showed increasing mortality across categories: 8% (low-risk), 33% (intermediate) and 57% (high-risk) (Table 4):

- < 9 points: 8% mortality (low-risk, *n* = 286).
- 9–16 points: 33% mortality (intermediate-risk, *n* = 164).
- ≥ 17 points: 57% mortality (high-risk, *n* = 60).
- ≥ 26 points: No patients had MELD-I ≥ 26 points (extreme-risk category, *n* = 0).

### Multivariable analysis

Multivariable logistic regression identified MELD-I as an independent predictor of in-hospital mortality (OR 1.07 per unit increase, 95% CI [1.03–1.11], *p* = 0.0002), followed by advanced age (OR 1.04 per year, 95% CI [1.01–1.05], *p* = 0.031) (Table 5). A 10-point increase in MELD-I corresponded to an approximately twofold increase in the odds of in-hospital mortality (OR 2.01, 95% CI 1.34–3.01) (Table 5). No relevant multicollinearity was observed among the included covariates. Variance inflation factors ranged from 1.08 to 1.72, remaining well below commonly accepted thresholds for problematic collinearity.

**Table 5:** Multivariable logistic regression for in-hospital mortality

MELD-I^2^	Odds Ratio	95% Confidence Interval	*p*-value^1^	
	1.07	1.03–1.11	0.0002	***
Age	1.03	1.01-0.105	0.031	*
Female sex	1.56	0.91–2.68	0.107	
History of MI^3^	1.58	0.76–3.27	0.228	
Ever-smoker	0.53	0.25–1.10	0.093	
Entry in zone 2^4^	1.74	0.74–4.06	0.207	
Entry in zone 3^5^	6.92	0.48–99.7	0.159	

^1^Pearson’s Chi-squared test; Wilcoxon rank sum test; Fisher’s exact test, ^2^Model for End-Stage Liver Disease (MELD-I), ^3^myocardial infarction in past medical history, ^4^ entry tear located in the aortic arch, ^5^ entry tear located in the descending thoracic aorta

### Discriminatory performance

**Fig. 7:**
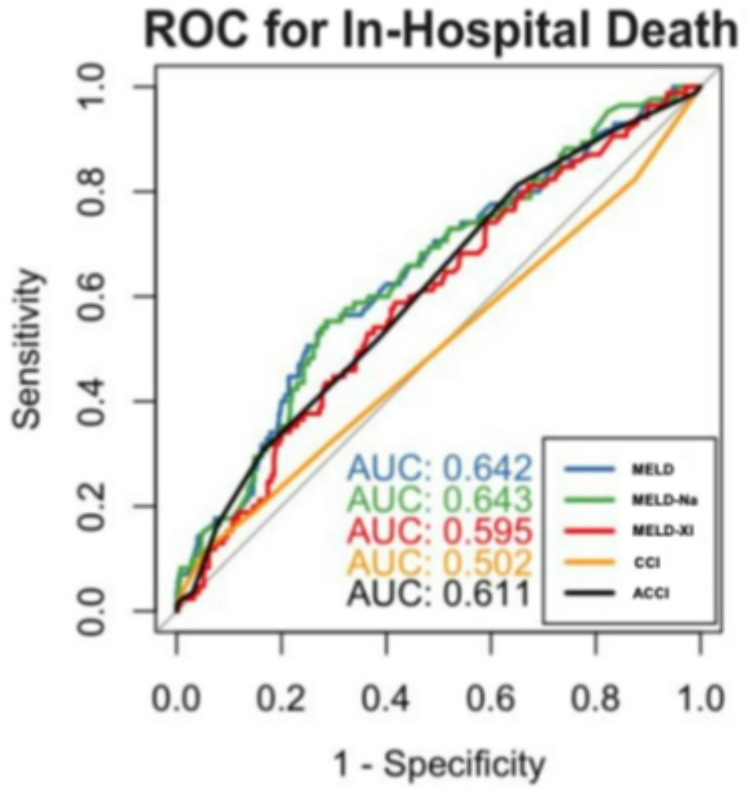
In-Hospital Mortality

ROC analysis demonstrated MELD-I had better discriminatory performance (AUC = 0.642, 95% CI [0.577–0.708]) (Fig. 7) than the Charlson Comorbidity Index (AUC = 0.502, 95% CI [0.435–0.568]). For 1-year mortality, age-adjusted CCI performed better (AUC = 0.662) than MELD variants (AUC = 0.590–0.591), suggesting different optimal scores for acute versus long-term prediction. However, the absolute difference in discrimination remained modest (ΔAUC = 0.14), indicating that MELD-I should be regarded as a complementary rather than stand-alone risk stratification tool. Bootstrap-corrected ROC analysis demonstrated only mi

nor deviations from the original AUC estimates, supporting the robustness of the observed discriminatory performance.

### Long-term survival

Kaplan-Meier 3-year survival curves stratified by MELD-I demonstrated significant separation (log-rank *p* = 0.00053, Figs. 1, 2, 3, 4, 5 and 6). Female patients showed worse long-term survival (*p* = 0.0052), whereas surgical approach did not influence outcomes (*p* = 0.7, Table 6).

**Table 6:** Characteristics by one-year mortality (112 Patients were excluded due to incomplete records *n* = 98 and loss of FU *n* = 14, *n* = 398 patients with complete FU)

Variable	Overall *n* = 398^1^	Survivors *n* = 279^1^	Non-Survivors *n* = 119^1^	*p*-value^2^
Male sex	274 (69%)	207 (74%)	67 (56%)	< 0.001
BMI^3^	26.6 (24.5, 29.7)	26.9 (24.5, 29.8)	26.6 (24.2, 29.4)	0.5
Age in years^4^	61 (52, 72)	58 (50, 68)	69 (61, 76)	< 0.001
History of MI^5^	36 (9.0%)	16 (5.7%)	20 (17%)	< 0.001
Ever-smoker^6^	87 (22%)	71 (25%)	16 (13%)	0.008
Diabetes	31 (7.8%)	20 (7.2%)	11 (9.2%)	0.5
Hyperlipidemia	91 (23%)	60 (22%)	31 (26%)	0.3
Hypertension^7^	261 (66%)	182 (65%)	79 (66%)	0.8
Active endocarditis	2 (0.5%)	0 (0%)	2 (1.7%)	0.089
Marfan syndrome	4 (1.0%)	4 (1.4%)	0 (0%)	0.3
Preoperative troponin^8^	22 (11, 51)	21 (10, 44)	25 (12, 64)	0.048
Entry zone				0.052
E1^9^	368 (92%)	262 (94%)	106 (89%)	
E2^10^	28 (7.0%)	17 (6.1%)	11 (9.2%)	
E3^11^	2 (0.5%)	0 (0%)	2 (1.7%)	
Malperfusion class				0.7
M0^12^	271 (68%)	191 (68%)	80 (67%)	
M1^13^	4 (1.0%)	2 (0.7%)	2 (1.7%)	
M2^14^	107 (27%)	76 (27%)	31 (26%)	
M3^15^	16 (4.0%)	10 (3.6%)	6 (5.0%)	
Total arch replacement	260 (65%)	184 (66%)	76 (64%)	0.7
Surgery duration in min^16^	340 (279, 415)	326 (270, 397)	358 (303, 474)	0.001
In-hospital death^17^	85 (21%)	4 (1.4%)	81 (68%)	< 0.001
Length of hospital stay^18^	14 (8, 21)	16 (12, 24)	2 (1, 10)	< 0.001
CCI including age^19^	4.00 (3.00, 5.00)	4.00 (3.00, 5.00)	5.00 (4.00, 6.00)	< 0.001
MELD-I^20^ ^7^	9.4 (6.3, 12.6)	8.5 (6.1, 11.4)	10.3 (7.2, 15.4)	0.004
MELD-Na^21^	9.7 (6.4, 13.3)	9.0 (6.2, 12.4)	10.8 (7.3, 15.5)	0.004
MELD-XI^22^	8.3 (5.6, 10.8)	8.2 (5.5, 10.7)	8.3 (5.7, 11.2)	0.6
MELD-I group^23^				0.003
1	216 (54%)	166 (59%)	50 (42%)	
2	135 (34%)	81 (29%)	54 (45%)	
3	47 (12%)	32 (11%)	15 (13%)	
MELD-Na group^24^				0.004
1	203 (51%)	157 (56%)	46 (39%)	
2	145 (36%)	88 (32%)	57 (48%)	
3	50 (13%)	34 (12%)	16 (13%)	
MELD-XI group^25^				> 0.9
1	276 (69%)	195 (70%)	81 (68%)	
2	116 (29%)	80 (29%)	36 (30%)	
3	6 (1.5%)	4 (1.4%)	2 (1.7%)	
Hospital stay group				< 0.001
< 14 days	223 (56%)	120 (43%)	103 (87%)	
> 14 days	175 (44%)	159 (57%)	16 (13%)	

^1^n, ^2^Pearson’s Chi-squared test; Wilcoxon rank sum test; Fisher’s exact test, ^3^body mass index, ^4^in years, ^5^myocardial infarction in past medical history, ^6^smoking active or in past medical history, ^7^arterial hypertension, ^8^in nanograms per liter, ^9^E1 = entry tear located in the ascending aorta, ^10^E2 = entry tear located in the aortic arch, ^11^E3 = entry tear located in the descending thoracic aorta, ^12^M0 = no malperfusion, ^13^M1 = coronary malperfusion, ^14^M2 = visceral or renal, malperfusion, ^15^M3 = peripheral or multiple-site malperfusion, ^16^in minutes, ^17^ (%), ^18^in days, ^19^unadjusted Charlson Comorbidity Index (CCI) in absolute CCI points, ^20^Model for End-Stage Liver Disease (MELD-I), ^21^MELD including sodium levels (MELD-Na), ^22^MELD excluding INR, ^23^MELD-I, ^24^MELD-Na, and ^25^MELD-XI were each grouped into three risk categories: scores < 9 = low risk, scores 9–16 = intermediate risk and scores > 17 = high risk. EuroSCORE II and STS scores could not be calculated due to incomplete documentation of required variables in this emergency setting

### Association between MELD scores and length of hospital stay

**Fig. 8:**
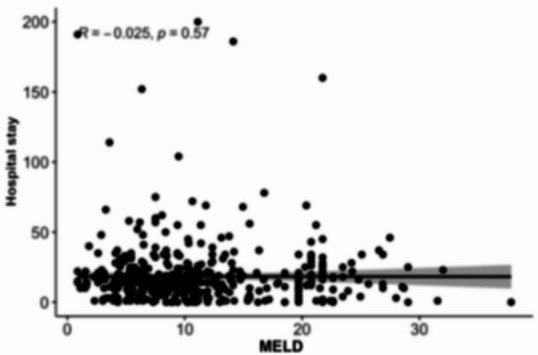
MELD-I

**Fig. 9:**
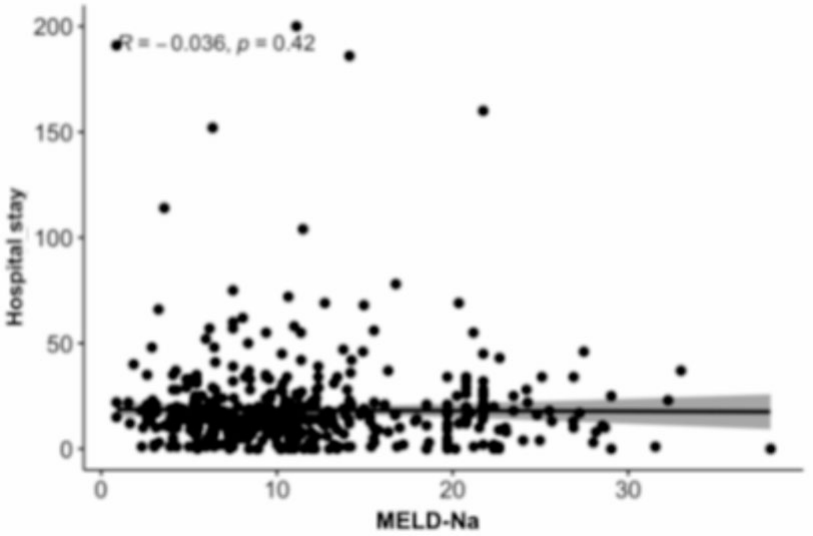
MELD-Na

**Fig. 10:**
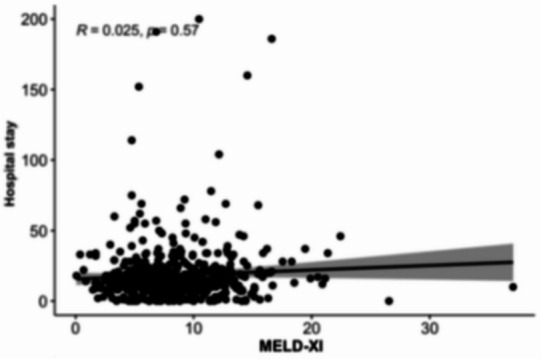
MELD-XI

**Fig. 11:**
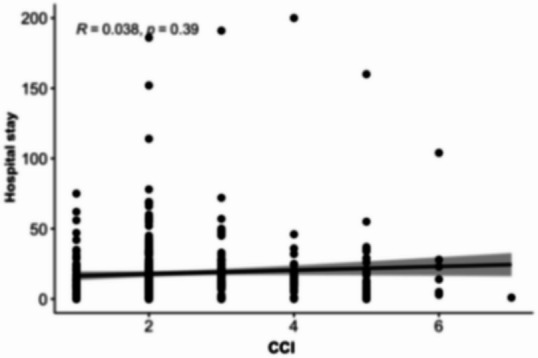
CCI

**Fig. 12:**
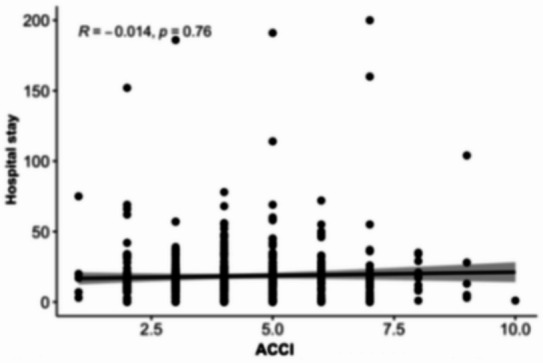
ACCI (age adjusted)

Scatterplot analyses (Figs. 8, 9, 10,11 and 12) demonstrated a positive association between increasing MELD-I, MELD-Na, MELD-XI, and CCI values and prolonged hospital stay. This relationship was most pronounced for MELD-I and MELD-Na, where higher scores were associated with greater dispersion toward longer hospitalization durations. In contrast, MELD-XI and CCI showed weaker correlations with length of stay. These findings support the concept that preoperative hepatorenal dysfunction contributes not only to mortality risk but also to postoperative recovery burden and resource utilization (Fig. 12).

### One year mortality

**Fig. 13:**
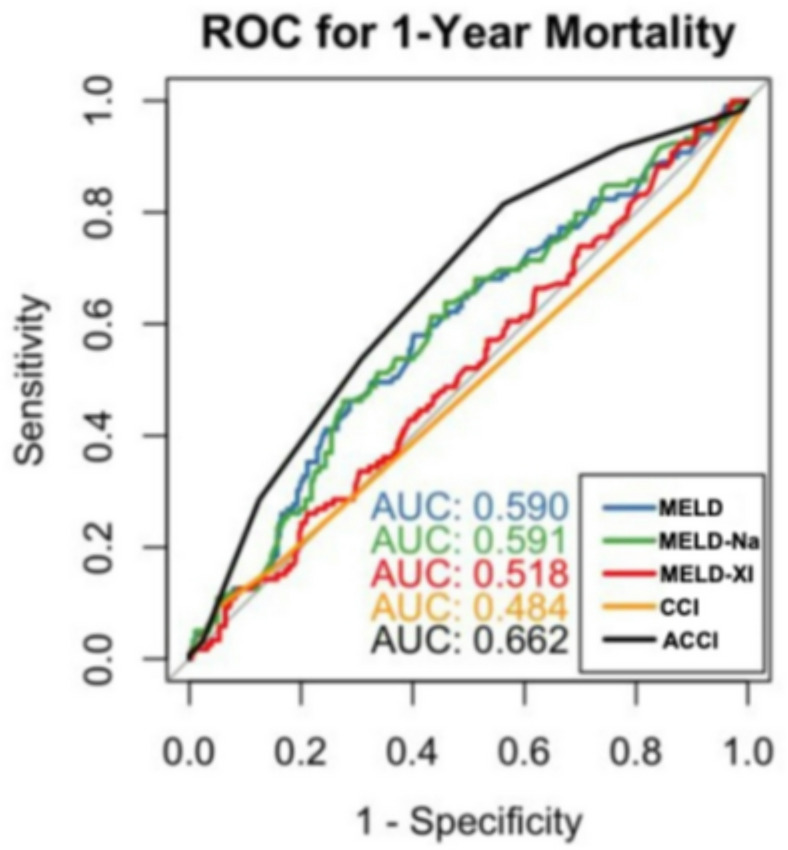
ROC analysis 1-year mortality

Of 510 patients, 112 were excluded due to incomplete records (*n* = 98) or loss to follow-up (*n* = 14), leaving 398 patients for 1-year analysis (Table 6). Among the 398 patients with complete one-year follow-up, 279 (70%) survived and 119 (30%) died within the first year after surgery. Non-survivors were significantly older than survivors (median age 69 [61–76] vs. 58 [50–68] years, *p* < 0.001) and were more frequently female (44% vs. 26%, *p* < 0.001). Preoperative troponin levels were higher in non-survivors (median 25 [12–64] vs. 21 [10–44], *p* = 0.048). A history of smoking was more prevalent in survivors (25% vs. 13%, *p* = 0.008). Operative duration was significantly longer in non-survivors compared to survivors (median 358 [303–474] vs. 326 [270–397] minutes, *p* = 0.001). In-hospital death accounted for 81 (68%) of the 119 one-year non-survivors. Hospital length of stay differed markedly, with a median of 16 (12–24) days in survivors versus 2 (1–10) days in non-survivors (*p* < 0.001). Other baseline characteristics, including BMI, diabetes, hyperlipidemia, arterial hypertension, aortic diameter, ASA classification, previous surgery, Marfan syndrome, malperfusion status, extent of arch replacement were not significantly associated with one-year mortality (Table 6; Fig. 13).

## Discussion

This single-center analysis of 510 patients undergoing surgery for acute type A aortic dissection (ATAAD) confirms that the preoperative MELD-I score independently predicts both in-hospital and long-term mortality. Compared to conventional comorbidity indices, MELD-I may improve acute risk stratification by capturing dynamic physiological derangements relevant in emergency settings. The stepwise increase in mortality across MELD-I categories supports its clinical relevance, although its overall discriminative performance remained moderate (AUC = 0.642). Given its objectivity and availability on admission, MELD-I may be considered as part of early triage decisions, especially under time-critical conditions.

### One-year mortality

Beyond sex-specific disparities, our analysis of one-year mortality (Table 6) revealed several additional predictors that warrant consideration. Increasing age was strongly associated with worse outcomes, consistent with prior reports identifying advanced age as a major determinant of postoperative mortality in ATAAD patients [1]. Similarly, higher preoperative troponin values predicted reduced survival, in line with studies demonstrating the prognostic impact of myocardial injury in ATAAD [7]. Operative duration was significantly longer among non-survivors, reflecting the well-documented association between prolonged surgery, increased perioperative complexity, and impaired outcomes [20]. Interestingly, a history of smoking was more prevalent among survivors, echoing the so-called “smoker’s paradox” described in acute cardiovascular disease, which may partly be explained by the younger age and lower comorbidity burden of smokers [21]. Prior publications have also demonstrated that elevated preoperative CRP levels are associated with worse outcomes in ATAAD [19]. Although CRP was not systematically available in our cohort and could therefore not be included in the present analysis, inflammatory activation may represent an additional pathophysiological pathway contributing to adverse postoperative outcomes. These findings highlight that, in addition to physiology-based scores such as MELD, conventional demographic and procedural variables continue to exert substantial influence on prognosis following ATAAD repair.

### Long-/Short term and prognostic value of MELD core beyond early postoperative phase

Although MELD was originally designed to predict short-term outcomes, our findings suggest that its components may retain prognostic value beyond the acute phase. Persistently elevated bilirubin or creatinine levels can indicate chronic hepatic or renal impairment, reflecting limited physiological reserve and increased susceptibility to long-term complications. However, the reduced predictive accuracy for 1-year mortality (AUC ~ 0.59) suggests that MELD is more effective in capturing acute risk rather than long-term trajectories. Combining MELD with chronic disease scores or postoperative variables may enhance its utility in long-term risk modeling. The Kaplan-Meier analysis beyond hospital discharge illustrates that the impact of preoperative organ dysfunction remains significant long after the acute event.

Although the intermediate-risk MELD-I group showed the lowest crude 3-year survival rate, whereas the high-risk group demonstrated a numerically slightly higher survival, this finding should be interpreted cautiously. The high-risk category included only 60 patients, resulting in less stable survival estimates and wider confidence intervals. In addition, early perioperative mortality was particularly pronounced in the highest MELD category, meaning that only a selected subgroup of patients survived the initial hospitalization and entered long-term follow-up. This survivor effect may partly explain why the long-term Kaplan-Meier estimates in the high-risk group appeared numerically better than those of the intermediate-risk group despite the overall stepwise increase in in-hospital mortality across MELD categories.

Patients exhibiting higher MELD scores, indicating profound hepatorenal impairment and coagulation disorders, carry an increased risk of mortality that may persist beyond the early postoperative phase. This demonstrates that MELD scores not only provide valuable prediction of early postoperative mortality but may also serve as important prognostic indicators for longer-term survival following repair of acute type A aortic dissection. However, the observed long-term survival estimates in the highest MELD category should be interpreted with caution because early perioperative mortality was substantial in this subgroup, leading to a survivor effect among patients who remained in long-term follow-up. Overall, our data suggest that MELD score variants may perform better than traditional comorbidity indices in this cohort, such as the Charlson score, in reflecting acute multisystem derangement and its sustained effect on survival. Incorporating these dynamic measures into risk stratification could enhance individualized perioperative management and post-discharge follow-up strategies.

### Association between MELD scores and length of hospital stay

The scatterplot analyses suggested that higher MELD-I and MELD-Na values were associated with prolonged hospitalization. This observation is clinically plausible, as patients with greater preoperative hepatorenal dysfunction and coagulation abnormalities may require more extensive postoperative monitoring, organ support, and rehabilitation. In contrast, MELD-XI and CCI demonstrated weaker associations with hospital stay, suggesting that these scores may be less sensitive to acute perioperative physiological burden. Although length of stay is influenced by multiple non-clinical factors, including discharge logistics and rehabilitation capacity, the observed relationship supports the broader relevance of MELD-based scores beyond mortality prediction alone.

### Physiological rationale and mechanistic interpretation

The prognostic value of the MELD score in ATAAD likely stems from its ability to quantify acute multisystem organ dysfunction—specifically, hepatorenal impairment as well as coagulation abnormalities—commonly observed in the early phases of dissection. Hemodynamic instability as well as systemic hypoperfusion frequently lead to ischemic hepatic injury as well as acute kidney injury. This, in turn, impairs detoxification, protein synthesis, as well as fluid/electrolyte homeostasis. Moreover, endothelial injury induced by the dissection process activates both pro-thrombotic as well as fibrinolytic pathways, resulting in a consumptive coagulopathy reflected by elevated INR values. These acute derangements are not adequately captured by traditional cardiac scores, which primarily assess chronic cardiovascular risk. In contrast, the MELD score reflects the real-time physiological state of the patient as well as provides valuable insight into their capacity to tolerate surgical intervention. These components reflect organ reserve and systemic impact, already discussed above. This pathophysiological framework supports the use of MELD for preoperative triage as well as early prognostic counseling.

### Mechanistic insights

The prognostic utility of the MELD score in ATAAD likely stems from its capacity to quantify key acute pathophysiological processes—namely, hepatorenal dysfunction as well as coagulopathy—that directly impact patient outcomes. ATAAD often causes hypoperfusion and hemodynamic instability, leading to hepatic and renal ischemia, which in turn impairs coagulation and elevates INR. Concurrently, renal hypoperfusion leads to acute kidney injury, impairing clearance of metabolic waste as well as contributing to fluid as well as electrolyte imbalances. Furthermore, the disruption of the vascular endothelium during dissection initiates a complex coagulative imbalance involving both hypercoagulability as well as consumptive coagulopathy. This multifaceted hemostatic disturbance is critical in influencing bleeding risk during surgical repair. The MELD score integrates key markers of hepatic, renal, and coagulative dysfunction, offering a snapshot of acute physiological instability. Therefore, MELD’s superiority in predicting short-term outcomes in ATAAD may be attributed to its sensitivity for detecting acute, life-threatening multisystem organ dysfunction that defines the emergent pathophysiology of the disease. While traditional comorbidity indices primarily quantify chronic disease burden, MELD captures acute organ dysfunction as well as physiologic instability more effectively than comorbidity-based scores [11, 12]. These derangements often reflect the severity of systemic inflammation, tissue hypoperfusion, as well as coagulative imbalance associated with the acute phase of dissection. The score’s stronger performance in predicting short-term, as opposed to long-term, outcomes suggests that MELD is particularly effective in capturing the acute physiological instability present at admission. Traditional comorbidity indices primarily reflect chronic disease burden, which may be less informative in the context of rapidly evolving hemodynamic instability typical of ATAAD.

### Comparison with existing literature and established risk scores

To contextualize these findings and assess their broader relevance, we compared MELD’s performance with established cardiac and aortic risk scores. Our findings are consistent with recent cardiac surgery literature that highlights the predictive value of the MELD score across a wide range of patient populations [10, 12, 13]. The observed stepwise increase in mortality—from 4.6% for MELD scores below 10 to 31.2% for MELD scores above 20—in mixed cardiac surgery cohorts closely parallels the risk stratification observed in our ATAAD-specific cohort [1]. However, the discriminatory performance in our study (AUC = 0.64) is more modest compared to the higher AUC values reported in some cardiac surgery studies (ranging from 0.75 to 0.81), suggesting that MELD’s predictive strength may vary depending on the specific surgical context and underlying pathophysiology. In contrast to ATAAD-specific scoring systems such as the GERAADA score (AUC 0.73–0.80) or broadly used cardiac risk models like EuroSCORE II (AUC 0.70–0.80), the MELD score offers the practical advantage of rapid bedside applicability based solely on routinely available laboratory parameters [15]. However, neither EuroSCORE II nor the STS score include laboratory-based markers of acute organ dysfunction, such as bilirubin, creatinine, or INR, which are highly relevant for prognosis in ATAAD [12]. Their dependence on extensive clinical and anatomical information further restricts feasibility in the emergency setting. This underscores the complementary role of MELD-based scores as physiology-driven tools for rapid risk assessment. EuroSCORE II, while achieving AUCs of 0.70–0.80 in elective cardiac surgery, may overestimate mortality in emergency ATAAD cases due to missing anatomical and procedural variables, EuroSCORE II could not be calculated in our cohort. In contrast, MELD offers rapid lab-based assessment with moderate accuracy (AUC 0.642), making it more suitable for acute triage. While the MELD score demonstrated a slightly lower AUC of 0.642 in our cohort, it provides a distinct advantage by objectively quantifying acute hepatorenal dysfunction and coagulopathy, thereby enabling more precise risk stratification of multisystem organ failure at presentation—a critical determinant of short-term outcomes in ATAAD [22]. This is particularly useful in emergencies, where rapid decisions are needed and full clinical data may be lacking. Although the GERAADA score has demonstrated superior discriminatory ability in recent validations, it relies on a broader set of variables—including detailed anatomical findings and intraoperative factors—which may limit its immediate utility during early triage or in centers with limited diagnostic resources. In this context, MELD may serve as a complementary tool for timely preoperative risk stratification when comprehensive data acquisition is not feasible. These findings highlight the complementary role of MELD-based scores as rapid physiology-driven tools for perioperative risk assessment in ATAAD.

### Sex-specific findings

The observed survival disadvantage among female patients may reflect anatomical, hormonal, or systemic differences, although residual confounding cannot be excluded (*p* = 0.0052). Because body surface area, aortic diameter, treatment delay, and hormonal status were not consistently available, no formal interaction analysis between sex and MELD score could be performed. Therefore, residual confounding cannot be excluded, and the observed sex-related differences should be interpreted cautiously. Future studies should examine hormonal status, vessel diameter, inflammatory markers, and potential effect modification to better understand sex-specific outcome differences influencing female outcomes in ATAAD [23]. Potential contributing-factors include sex-specific variations in aortic anatomy, such as smaller vessel diameters relative to body surface area, hormonal influences on vascular integrity, as well as differences in inflammatory or thrombotic response. Emerging long-term data suggest that female patients may face diagnostic delays and underutilization of surgery, potentially contributing to poorer outcomes. Further research is urgently needed to elucidate the underlying mechanisms as well as, more significantly, to identify modifiable factors that could improve outcomes in this patient subgroup. Addressing such disparities may ultimately enhance individualized treatment strategies as well as ensure equitable care in the management of ATAAD.

### Clinical implementation considerations

Higher MELD-I scores (≥ 17 points) were associated with substantially increased in-hospital mortality (57%). These high-risk patients may require particularly aggressive perioperative management. While an extreme-risk category (MELD-I ≥ 26) was theoretically defined, no patients in our cohort reached this threshold (*n* = 0). Nevertheless, the overall moderate discriminatory power of the MELD score highlights its limitations as a standalone prognostic instrument. These findings support its use not as an isolated decision-making tool, but rather as an integral component of a broader, multimodal risk assessment framework that also incorporates clinical judgment, anatomical considerations, and dynamic perioperative parameters. MELD can thus support early triage, resource allocation, and patient counseling—especially in time-sensitive situations.

### Limitations

This study has several important limitations that must be acknowledged:

1. Retrospective, single-center design introduces potential selection bias and limits generalizability.
2. Extended inclusion period (2000–2019) encompasses major advances in surgical and critical care, which may confound temporal trends.
3. Incomplete data for EuroSCORE II and STS precluded direct comparison with widely used cardiac risk models.
4. Moderate discriminatory performance limits MELD’s role as a standalone predictor; laboratory turnaround times may constrain real-time applicability in resource-limited settings.
5. Absence of external validation necessitates multicenter prospective studies to confirm reproducibility.
6. Missing ICU-specific scores (SOFA, SAPS II) restricts contextualization of MELD’s performance relative to established organ failure indices.

Clinical context:

Nonetheless, the clinical urgency of ATAAD demands tools that are immediately applicable, even if their predictive precision is limited.

The emergent nature of acute type A aortic dissection (ATAAD) limits the opportunity for comprehensive preoperative risk assessment, rendering MELD’s immediate availability from routine laboratory parameters potentially valuable despite its moderate predictive accuracy. While MELD relies on commonly obtained markers such as bilirubin, creatinine, and INR, their availability in emergency settings may vary. In particular, turnaround times for INR and bilirubin can delay real-time scoring in hemodynamically unstable patients. Nevertheless, all MELD components are typically accessible within a few hours in tertiary care centers, supporting the feasibility of MELD-based risk stratification for early triage in most acute care environments [24].

## Conclusion

The preoperative MELD-I score is an independent predictor of both in-hospital and long-term mortality following surgical repair of acute type A aortic dissection (ATAAD). Although its overall discriminatory performance is moderate, MELD-I showed better discriminatory performance in this cohort traditional comorbidity indices and enables rapid, objective risk stratification based on admission laboratory values. The clear stepwise increase in mortality across MELD categories highlights its clinical relevance for perioperative triage, particularly in resource-limited or time-critical settings. By providing a physiology-based assessment of multisystem organ dysfunction—an aspect often overlooked by cardiac-specific models such as EuroSCORE II—MELD represents a practical adjunct to multimodal risk assessment strategies. It should not replace anatomical or procedural risk models but rather complement them within a comprehensive framework. Future research should focus on prospective multicenter validation, integration with ATAAD-specific scores such as GERAADA, and exploration of sex-specific survival disparities. Additionally, MELD’s potential role in guiding perioperative management warrants further investigation to enhance individualized care in this high-risk population.

## Data Availability

The data underlying this study were obtained retrospectively from clinical records and contain sensitive personal health information. Due to privacy regulations and ethical considerations, the raw data cannot be made publicly available. Researchers with a legitimate interest may request access to anonymized or aggregated data, subject to approval by the responsible ethics committee and in compliance with applicable data protection laws.
